# Supplementary material for: CHD4 regulates platinum sensitivity through MDR1 expression in ovarian cancer: A potential role of CHD4 inhibition as a combination therapy with platinum agents
Source: PLoS One. 2021 Jun 23;16(6):e0251079. doi: 10.1371/journal.pone.0251079 (PMC8221472; doi:10.1371/journal.pone.0251079)
Supplement: S1 Fig — a) Survival comparison between the two groups divided at median CHD4 mRNA expression. No statistically significant difference was observed. b) Representative examples positive and negative for CHD4 immunohistochemistry. A sample was considered to be CHD4 positive if more than 50% of the cancer cell nuclei were stained. A summary of CHD4 immunohistochemistry among the cases shown in Fig 1B. p = 0.28 by Fisher’s exact test. (DOCX) [file pone.0251079.s001.docx]

**Supporting Information**


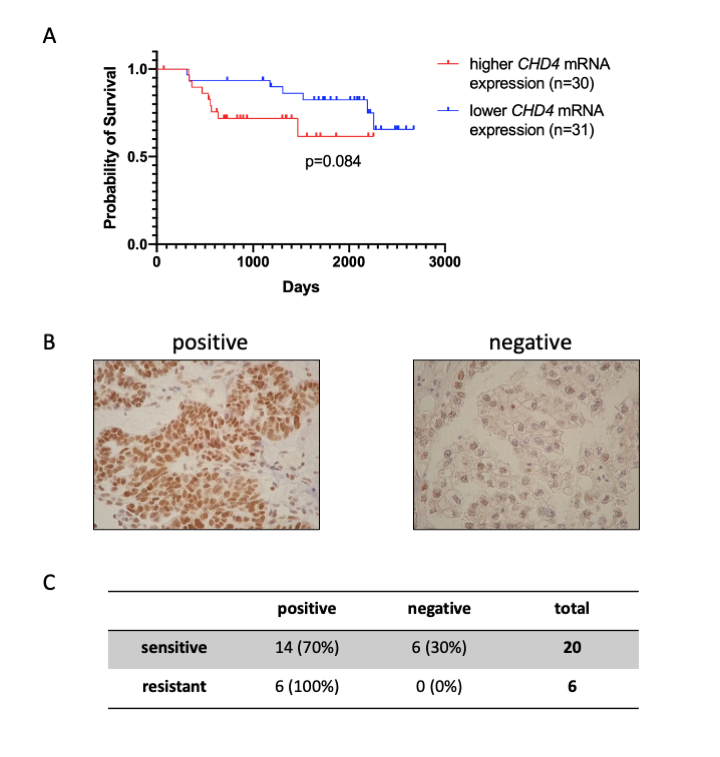


**S1 Fig. Correlation between CHD4 expression and clinical outcome**

1. Survival comparison between the two groups divided at median CHD4 mRNA expression. No statistically significant difference was observed.
2. Representative examples positive and negative for CHD4 immunohistochemistry. A sample was considered to be CHD4 positive if more than 50% of the cancer cell nuclei were stained.
3. A summary of CHD4 immunohistochemistry among the cases shown in Fig 1B. p=0.28 by Fisher’s exact test.
