# Supplementary material for: CHD4 regulates platinum sensitivity through MDR1 expression in ovarian cancer: A potential role of CHD4 inhibition as a combination therapy with platinum agents
Source: PLoS One. 2021 Jun 23;16(6):e0251079. doi: 10.1371/journal.pone.0251079 (PMC8221472; doi:10.1371/journal.pone.0251079)
Supplement: S2 Fig — a) Quantitative PCR to compare CHD4 mRNA expression across cell lines. mRNA expression was normalized to GAPDH mRNA expression. b) Western blotting to compare CHD4 protein expression across cell lines. (DOCX) [file pone.0251079.s002.docx]

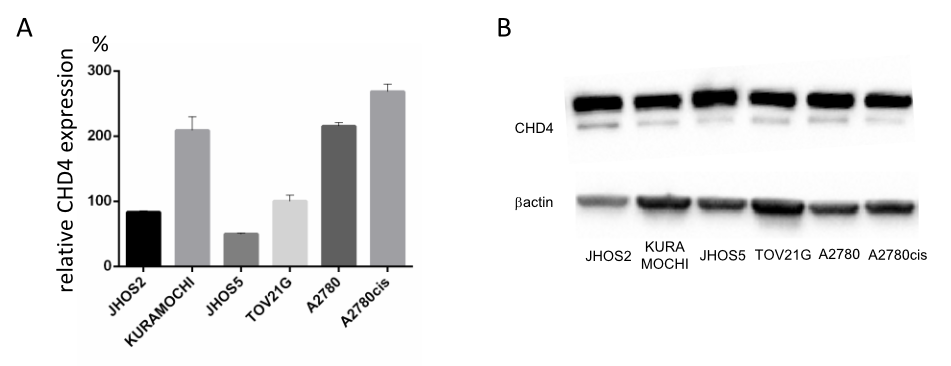


**S2 Fig. CHD4 expression across ovarian cancer cell lines**

1. Quantitative PCR to compare CHD4 mRNA expression across cell lines. mRNA expression was normalized to GAPDH mRNA expression.
2. Western blotting to compare CHD4 protein expression across cell lines.
