# Supplementary material for: CHD4 regulates platinum sensitivity through MDR1 expression in ovarian cancer: A potential role of CHD4 inhibition as a combination therapy with platinum agents
Source: PLoS One. 2021 Jun 23;16(6):e0251079. doi: 10.1371/journal.pone.0251079 (PMC8221472; doi:10.1371/journal.pone.0251079)
Supplement: S3 Fig — After 72 hours of siRNA transfection, TOV21G, JHOC5, and KURAMOCHI were treated with 0.5, 1, 2, 5, 10 μM of cisplatin. Relative cell viability was determined in 72 hours of cisplatin treatment and compared between CHD4 knockdown and control groups. *p < 0.05; **p < 0.01; ***p < 0.001. siCTRL; negative control siRNA. (DOCX) [file pone.0251079.s003.docx]

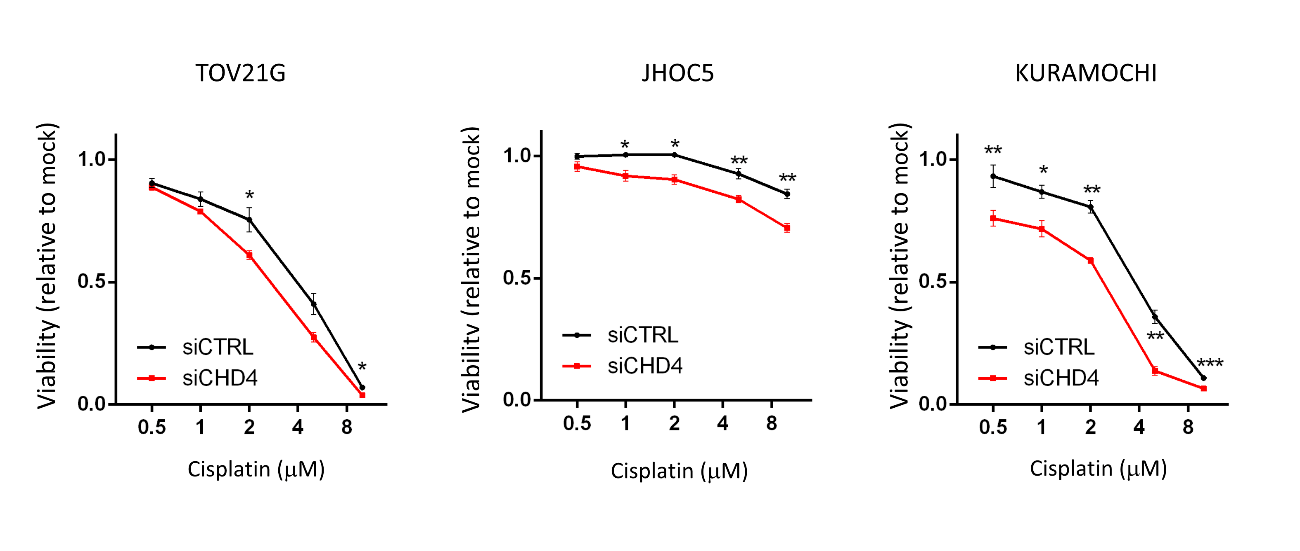


**S3 Fig. Cell viability assay with cisplatin treatment under CHD4 knockdown.**

After 72 hours of siRNA transfection, TOV21G, JHOC5, and KURAMOCHI were treated with 0.5, 1, 2, 5, 10 μM of cisplatin. Relative cell viability was determined in 72 hours of cisplatin treatment and compared between CHD4 knockdown and control groups. *p < 0.05; **p < 0.01; ***p < 0.001. siCTRL; negative control siRNA
