# Supplementary material for: CHD4 regulates platinum sensitivity through MDR1 expression in ovarian cancer: A potential role of CHD4 inhibition as a combination therapy with platinum agents
Source: PLoS One. 2021 Jun 23;16(6):e0251079. doi: 10.1371/journal.pone.0251079 (PMC8221472; doi:10.1371/journal.pone.0251079)
Supplement: S4 Fig — Twenty-four hours after the transection of CHD4 siRNA or negative control siRNA, TOV21G cells were harvested and re-plated on a well of 6-well plate. The cells were treated with 5 μM of cisplatin or vehicle 24 hours after re-plating and colonies were stained 72 hours after cisplatin treatment. siCTRL, negative control siRNA. (DOCX) [file pone.0251079.s004.docx]

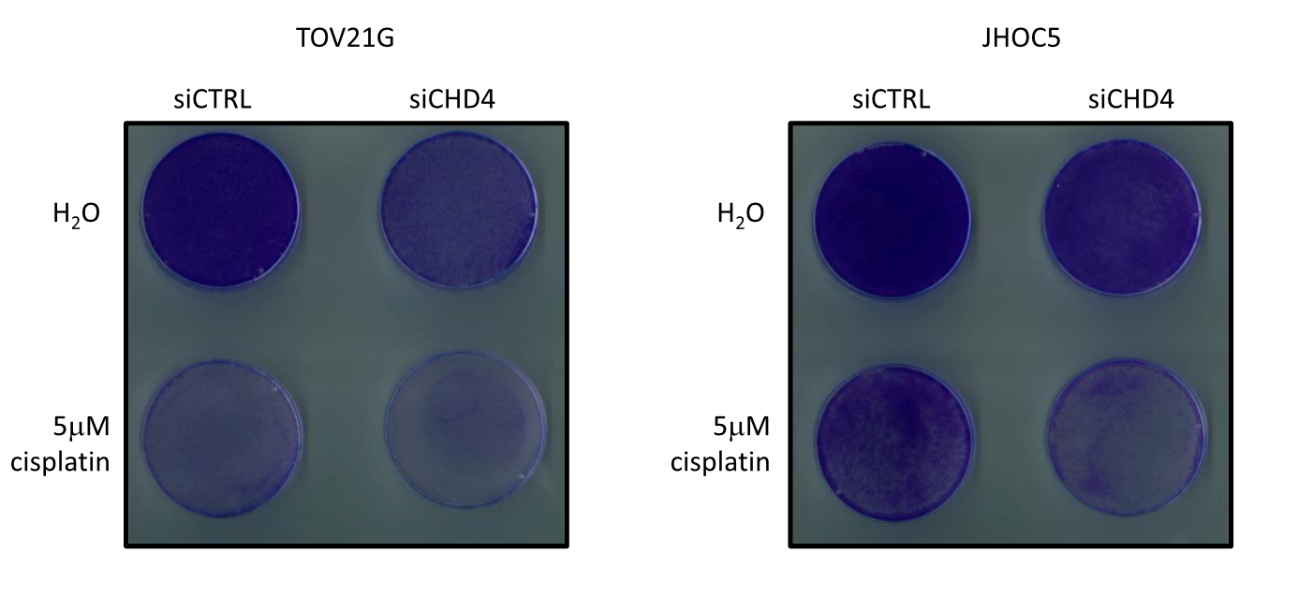


**S4 Fig. Clonogenic assay to assess the influence of CHD4 knockdown on cisplatin sensitivity**

Twenty-four hours after the transection of CHD4 siRNA or negative control siRNA, TOV21G cells were harvested and re-plated on a well of 6-well plate. The cells were treated with 5 μM of cisplatin or vehicle 24 hours after re-plating and colonies were stained 72 hours after cisplatin treatment. siCTRL, negative control siRNA
