# Supplementary material for: CHD4 regulates platinum sensitivity through MDR1 expression in ovarian cancer: A potential role of CHD4 inhibition as a combination therapy with platinum agents
Source: PLoS One. 2021 Jun 23;16(6):e0251079. doi: 10.1371/journal.pone.0251079 (PMC8221472; doi:10.1371/journal.pone.0251079)
Supplement: S5 Fig — Following 24 hours after transfection of control and CHD4 siRNA, OSE2 and OSE4 cells were treated by cisplatin for 72 hours, and then cell viability was assessed using Cell counting kit 8. IC50 of control and CHD4 knocked-down cells are 7.54 μM and 5.65 μM, respectively, in OSE2, and 2.37 μM and 2.23μM, respectively, in OSE4. IC50 values were compared between control and CHD4 knocked-down cells using Student’s t-test. The knockdown efficacy of CHD4 was validated by western blotting. siCTRL, negative control siRNA. (DOCX) [file pone.0251079.s005.docx]

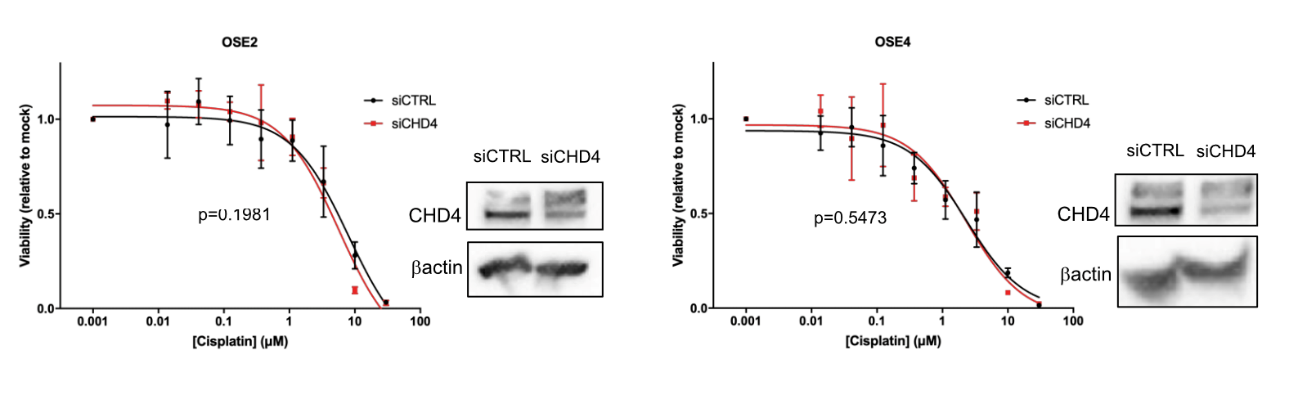


**S5 Fig. Dose-response curves to cisplatin treatment under CHD4 knockdown in OSE2 and OSE4**

Following 24 hours after transfection of control and CHD4 siRNA, OSE2 and OSE4 cells were treated by cisplatin for 72 hours, and then cell viability was assessed using Cell counting kit 8.

IC50 of control and CHD4 knocked-down cells are 7.54 µM and 5.65 µM, respectively, in OSE2, and 2.37 µM and 2.23µM, respectively, in OSE4. IC50 values were compared between control and CHD4 knocked-down cells using Student’s *t*-test. The knockdown efficacy of CHD4 was validated by western blotting. siCTRL, negative control siRNA
