# Supplementary material for: CHD4 regulates platinum sensitivity through MDR1 expression in ovarian cancer: A potential role of CHD4 inhibition as a combination therapy with platinum agents
Source: PLoS One. 2021 Jun 23;16(6):e0251079. doi: 10.1371/journal.pone.0251079 (PMC8221472; doi:10.1371/journal.pone.0251079)
Supplement: S6 Fig — siRNA targeting CHD4 or negative control was transfected to two ovarian cancer cells derived from clear cell carcinoma subtype (TOV21G, JGOC5) and two high-grade serous subtype cells (KURAMOCHI, JGOS2). Cell viability was assessed 72, 96, and 120 hours after transfection. * represents p ≤ 0.05; Abbreviations: siCTRL, negative control siRNA; CCC, clear cell carcinoma; HGSC, high-grade serous carcinoma. (DOCX) [file pone.0251079.s006.docx]

**S6 Fig.** **The influence of CHD4 knockdown on cell proliferation in ovarian cancer cells** siRNA targeting CHD4 or negative control was transfected to two ovarian cancer cells derived from clear cell carcinoma subtype (TOV21G, JGOC5) and two high-grade serous subtype cells (KURAMOCHI, JGOS2). Cell viability was assessed 72, 96, and 120 hours after transfection.

* represents p ≤ 0.05; Abbreviations: siCTRL, negative control siRNA; CCC, clear cell carcinoma; HGSC, high-grade serous carcinoma
