# Supplementary material for: CHD4 regulates platinum sensitivity through MDR1 expression in ovarian cancer: A potential role of CHD4 inhibition as a combination therapy with platinum agents
Source: PLoS One. 2021 Jun 23;16(6):e0251079. doi: 10.1371/journal.pone.0251079 (PMC8221472; doi:10.1371/journal.pone.0251079)
Supplement: S7 Fig — TOV21G, JHOC5, KURAMOCHI, and JHOS2 cells were treated with up to 30 μM of ED2-AD101. Each curve represents the mean of the triplicates. (DOCX) [file pone.0251079.s007.docx]

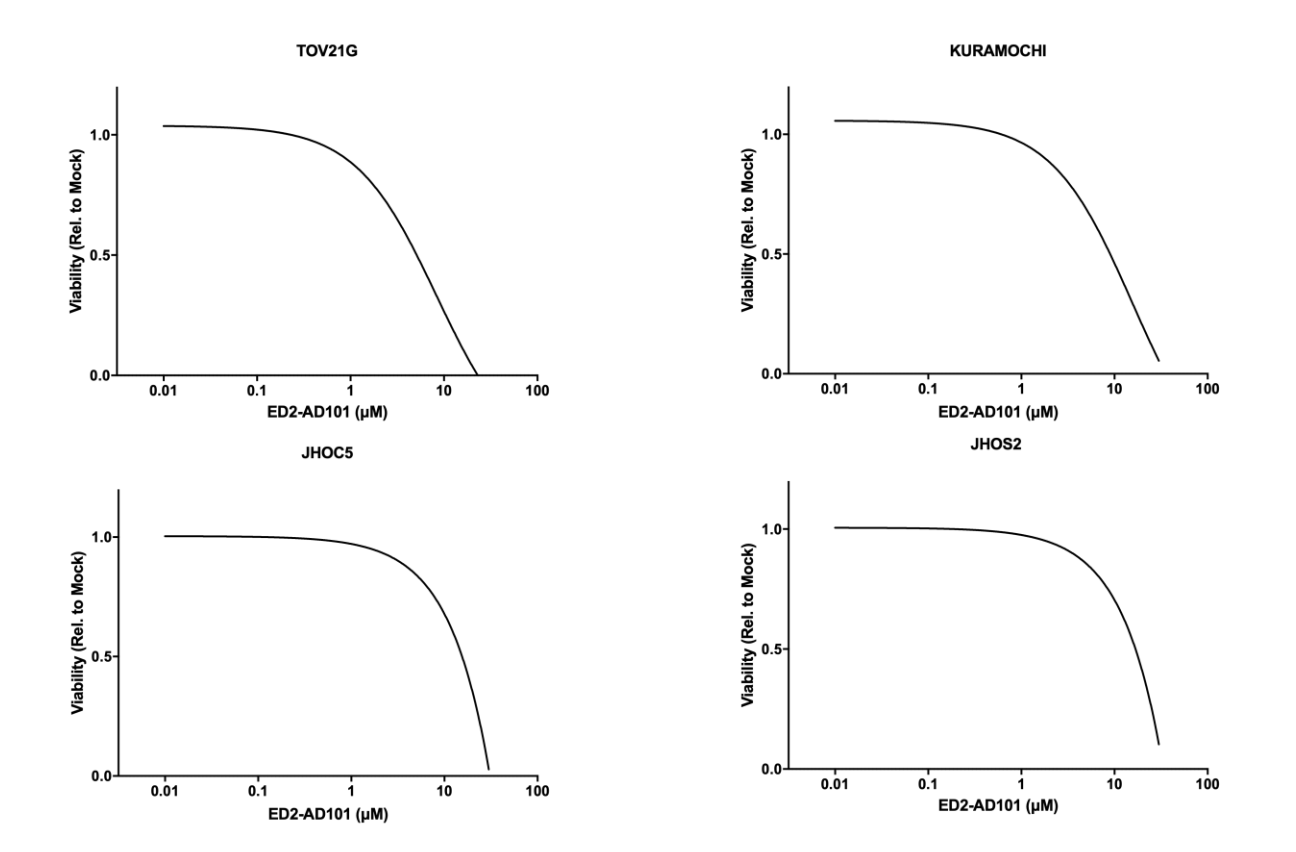


**S7 Fig. Dose response results of ovarian cancer cell lines to ED2-AD101**

TOV21G, JHOC5, KURAMOCHI, and JHOS2 cells were treated with up to 30 μM of ED2-AD101. Each curve represents the mean of the triplicates.
