# Supplementary material for: CHD4 regulates platinum sensitivity through MDR1 expression in ovarian cancer: A potential role of CHD4 inhibition as a combination therapy with platinum agents
Source: PLoS One. 2021 Jun 23;16(6):e0251079. doi: 10.1371/journal.pone.0251079 (PMC8221472; doi:10.1371/journal.pone.0251079)
Supplement: S8 Fig — 72 hours after the transfection with CHD4 siRNA or negative control siRNA, TOV21G cells were treated with 5 μM of cisplatin or vehicle for 24 hour and subjected to flow cytometry. The mean of three independent assays were documented. siCTRL; negative control siRNA. (DOCX) [file pone.0251079.s008.docx]

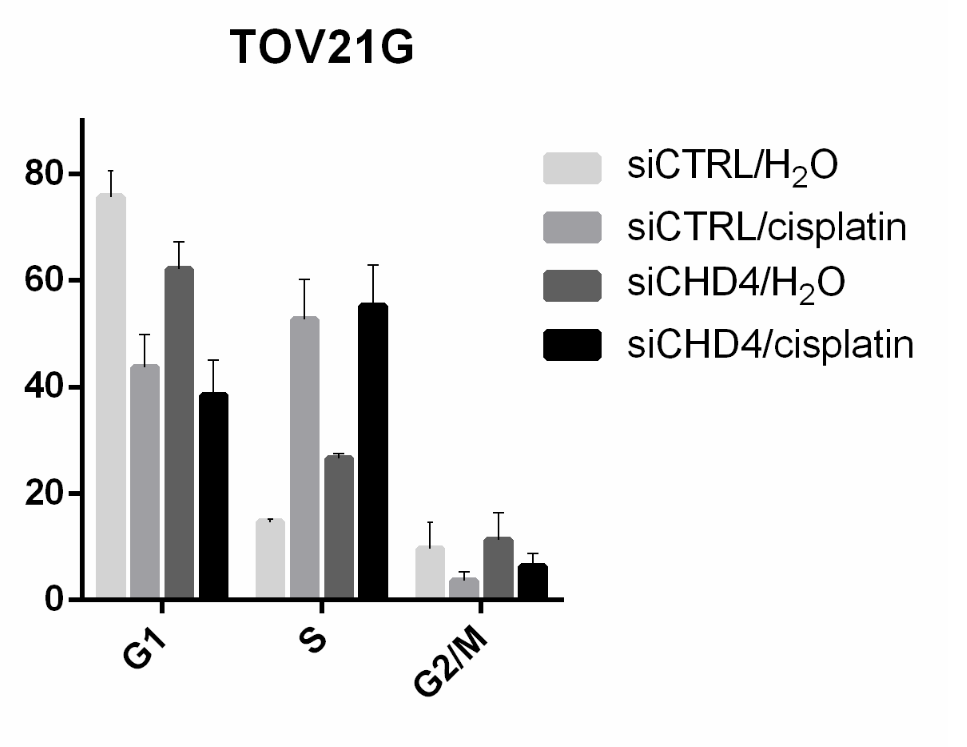


**S8 Fig. Influence of cisplatin treatment and CHD4 knockdown on cell cycle**

72 hours after the transfection with CHD4 siRNA or negative control siRNA, TOV21G cells were treated with 5 μM of cisplatin or vehicle for 24 hour and subjected to flow cytometry. The mean of three independent assays were documented. siCTRL; negative control siRNA
