# Supplementary material for: CHD4 regulates platinum sensitivity through MDR1 expression in ovarian cancer: A potential role of CHD4 inhibition as a combination therapy with platinum agents
Source: PLoS One. 2021 Jun 23;16(6):e0251079. doi: 10.1371/journal.pone.0251079 (PMC8221472; doi:10.1371/journal.pone.0251079)
Supplement: S9 Fig — (A) RAD51 and p21 expression 72 hours after siRNA transfection in JHOC5. Relative CHD4 expression normalized by the expression of β-actin was also determined by densitometry and demonstrated in the figure. (B) Quantification of RAD51 expression in TOV21G under CHD4 knockdown in comparison with control siRNA transfection. The bands were quantified by densitometry and CHD4 expression was normalized by β-actin expression. The data in the figure represents the mean of three independent assays. Statistical comparison was performed by one-sample t test. (C) MDR1 expression 72 hours after siRNA transfection in JHOC5 and JHOS2. Data were the representative of at least two independent assays. abbreviations: siCTRL; negative control siRNA. n.s.; not significant. (DOCX) [file pone.0251079.s009.docx]

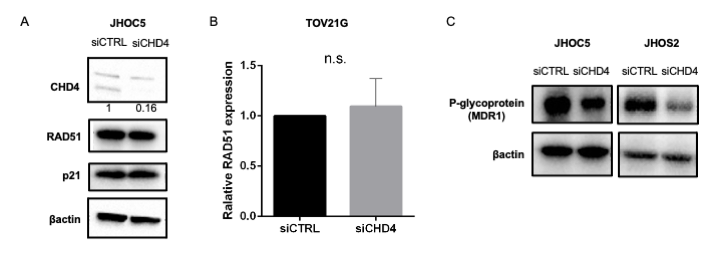


**S9 Fig.** **RAD51, p21, and p-glycoprotein expression under CHD4 knockdown**

(A RAD51 and p21 expression 72 hours after siRNA transfection in JHOC5. Relative CHD4 expression normalized by the expression of β-actin was also determined by densitometry and demonstrated in the figure. (B) Quantification of RAD51 expression in TOV21G under CHD4 knockdown in comparison with control siRNA transfection. The bands were quantified by densitometry and CHD4 expression was normalized by β-actin expression. The data in the figure represents the mean of three independent assays. Statistical comparison was performed by one-sample t test. (C) MDR1 expression 72 hours after siRNA transfection in JHOC5 and JHOS2. Data were the representative of at least two independent assays.

abbreviations: siCTRL; negative control siRNA. n.s.; not significant
