# Supplementary figures and images for: CHD4 regulates platinum sensitivity through MDR1 expression in ovarian cancer: A potential role of CHD4 inhibition as a combination therapy with platinum agents
Source: PLoS One. 2021 Jun 23;16(6):e0251079. doi: 10.1371/journal.pone.0251079 (PMC8221472; doi:10.1371/journal.pone.0251079)

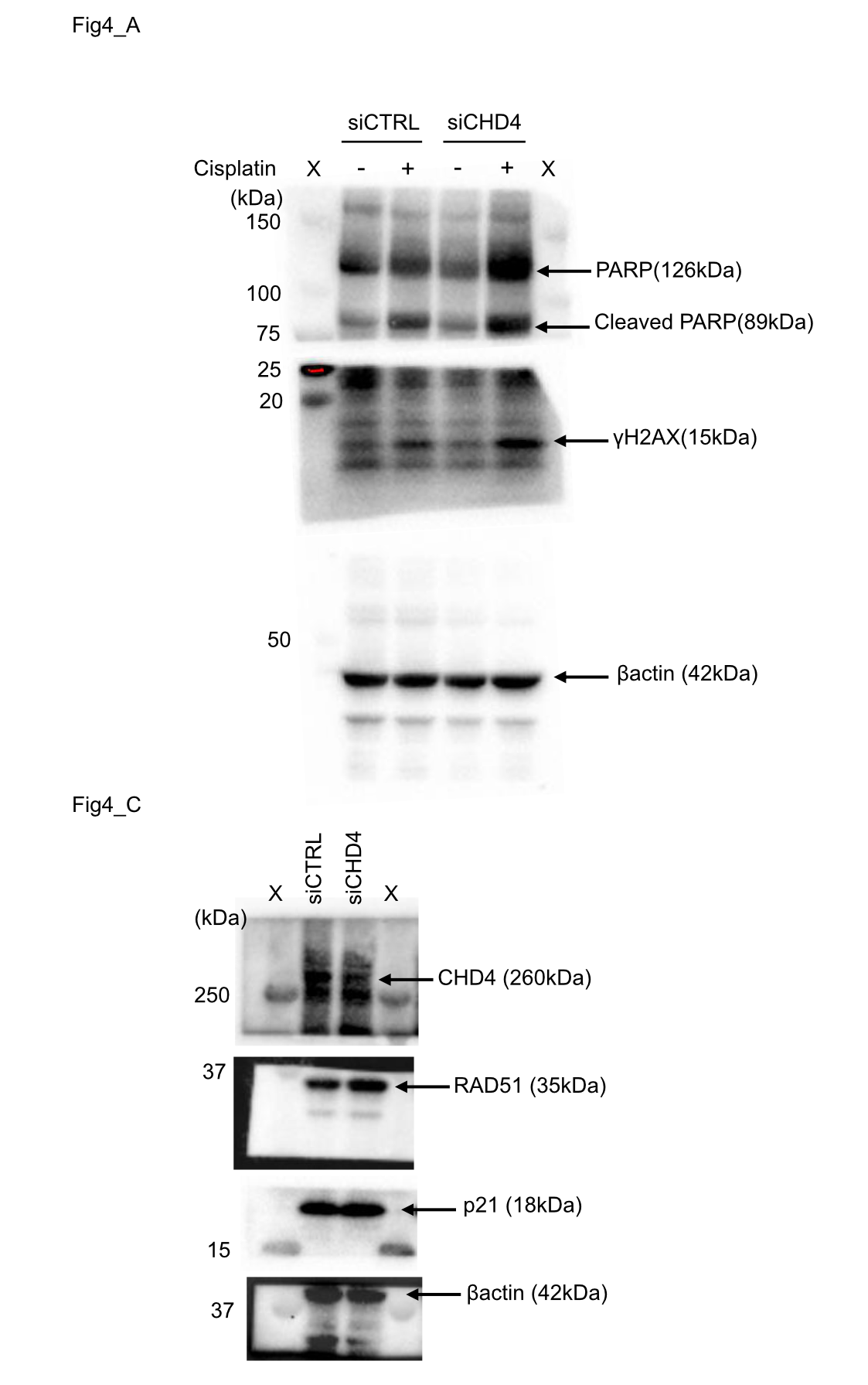
S10 Fig. Raw data of Western blotting for the figures and the supplementary information.


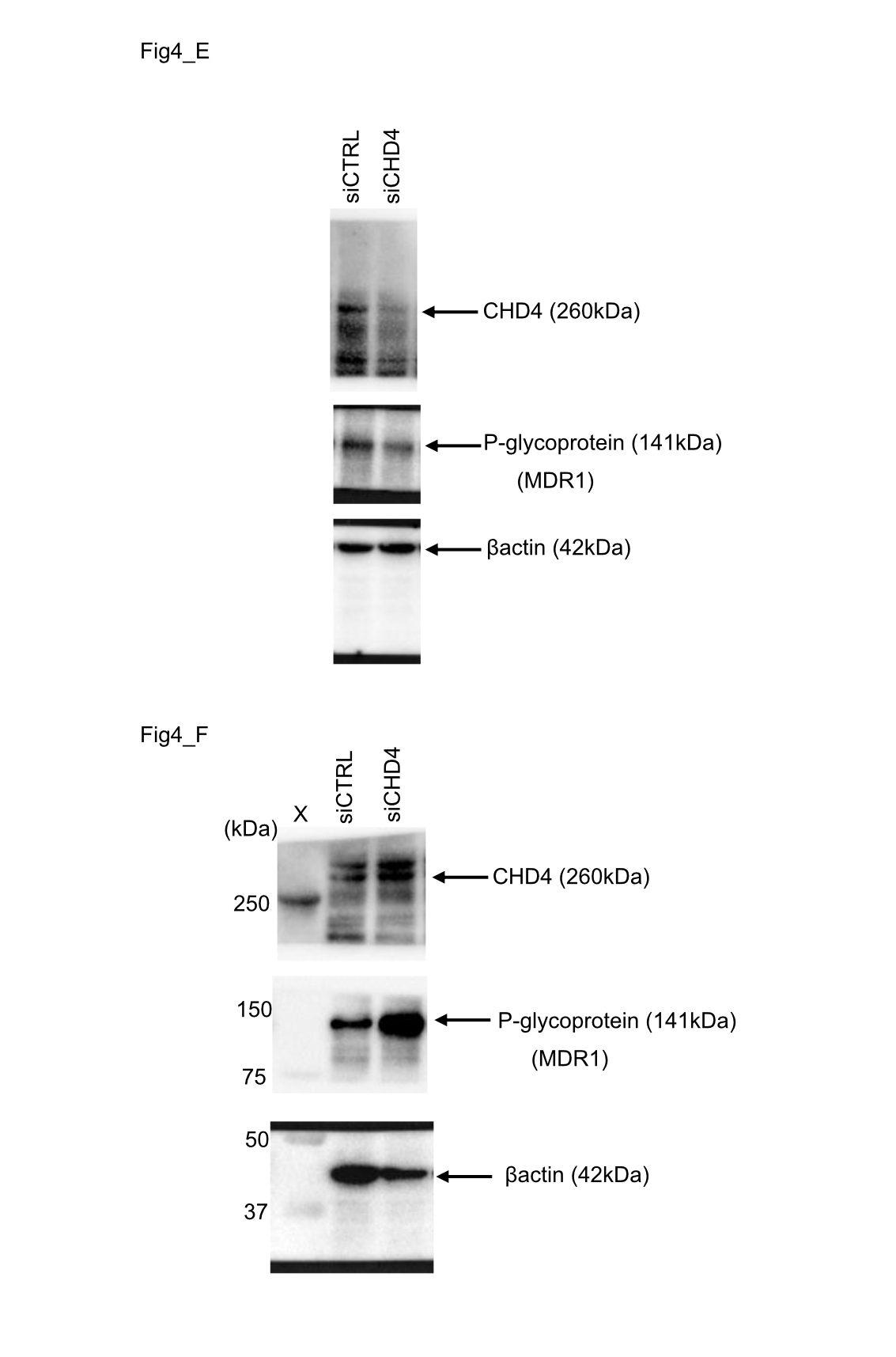


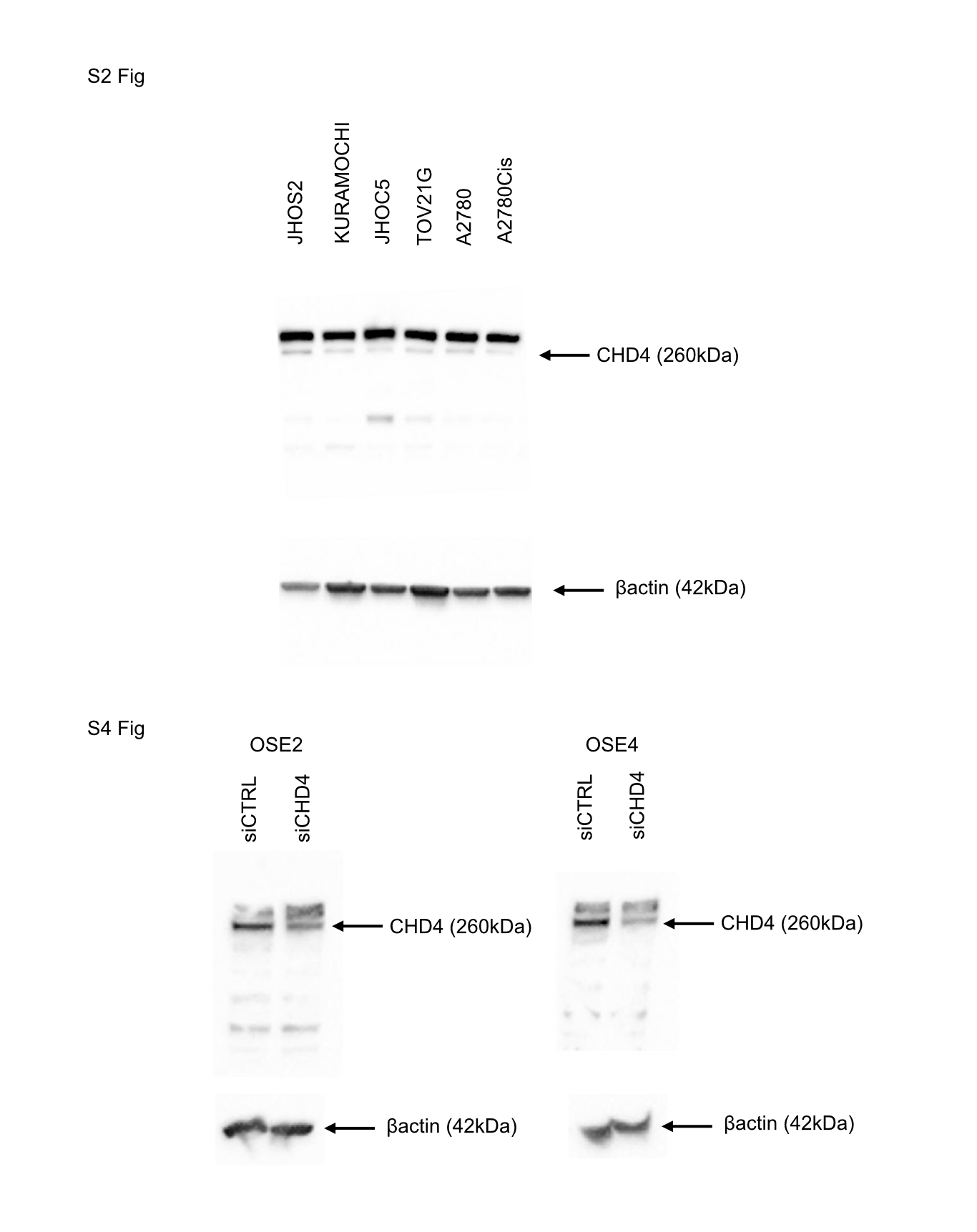


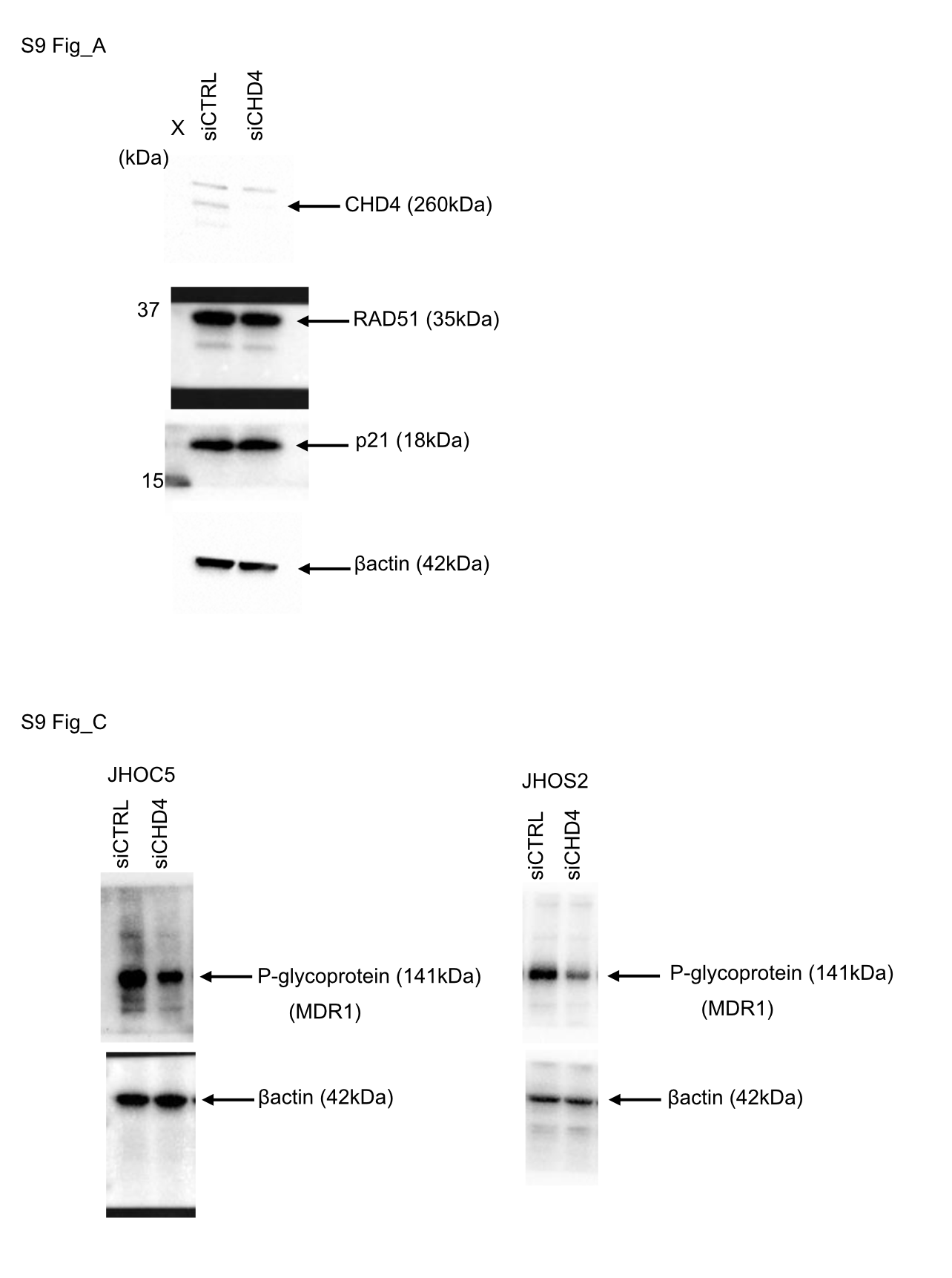

Supplement: S10 Fig — (DOCX) [file pone.0251079.s010.docx]
