# Supplementary material for: CHD4 regulates platinum sensitivity through MDR1 expression in ovarian cancer: A potential role of CHD4 inhibition as a combination therapy with platinum agents
Source: PLoS One. 2021 Jun 23;16(6):e0251079. doi: 10.1371/journal.pone.0251079 (PMC8221472; doi:10.1371/journal.pone.0251079)
Supplement: S1 Table — (DOCX) [file pone.0251079.s011.docx]

| Cells | IC50: siCTRL (mean ± SD) | IC50: siCHD4 (mean ± SD) |
| --- | --- | --- |
| TOV21G | 9.51 ± 1.097 | 1.96 ± 0.328 |
| JHOC5 | 8.12 ± 3.169 | 1.32 ± 0.740 |
| KURAMOCHI | 7.214 ± 1.135 | 3.224 ± 0.831 |
| JHOS2 | 5.996 ± 1.102 | 3.385 ± 0.782 |

**S table. IC50 determined by the dose-response results shown in Figure 2**

siCTRL; negative controls siRNA
